# Calculation of IFT in porous media in the presence of different gas and normal alkanes using the modified EoS

**DOI:** 10.1038/s41598-023-35320-3

**Published:** 2023-05-18

**Authors:** Sareh Hamidpour, Ali Safaei, Yousef Kazemzadeh, Atefeh Hasan-Zadeh, Azizollah Khormali

**Affiliations:** 1 Enhanced Oil Recovery Research Center, School of Chemical and Petroleum Engineering, Shiraz University, Shiraz, Iran; 2 Department of Petroleum Engineering, Gas and Petrochemical Engineering, Faculty of Petroleum, Persian Gulf University, Bushehr, 75169-13817 Iran; 3 Fouman Faculty of Engineering, College of Engineering, University of Tehran, P.O.Box 43581-39115, Guilan, Iran; 4 Department of Chemistry, Faculty of Basic Sciences and Engineering, Gonbad Kavous University, Gonbad Kavous, Iran

**Keywords:** Engineering, Chemical engineering, Crude oil, Natural gas, Petrol

## Abstract

Gas injection can increase oil recovery because the gas–oil interfacial tension is less than the water–oil interfacial tension (IFT) and tends to zero in the miscibility state. However, little information has been provided on the gas–oil movement and penetration mechanisms in the fracture system at the porosity scale. The IFT of oil and gas in the porous medium changes and can control oil recovery. In this study, the IFT and the minimum miscibility pressure (MMP) are calculated using the cubic Peng-Robinson equation of state that has been modified using the mean pore radius and capillary pressure. The calculated IFT and MMP change with the pore radius and capillary pressure. To investigate the effect of a porous medium on the IFT during the injection of CH_4_, CO_2_, and N_2_ in the presence of n-alkanes and for validation, measured experimental values in references have been used. According to the results of this paper, changes in IFT vary in terms of pressure in the presence of different gases and, the proposed model has good accuracy for measuring the IFT and the MMP during the injection of hydrocarbon gases and CO_2_. In addition, as the average radius of the pores gets smaller, the interfacial tension tends to lower values. This effect is different with increasing the mean size of interstice in two different intervals. In the first interval, i.e. the R_p_ from 10 to 5000 nm, the IFT changes from 3 to 10.78 mN/m and in the second interval, i.e. the R_p_ from 5000 nm to infinity, the IFT changes from 10.78 to 10.85 mN/m. In other words, increasing the diameter of the porous medium to a certain threshold (i.e. 5000 nm) increases the IFT. As a rule, changes in IFT affected by exposure to a porous medium affect the values of the MMP. In general, IFT decreases in very fine porous media, causing miscibility at lower pressures.

## Introduction

During the injection of gas into the porous medium saturated with oil, the movement can change from immiscible to almost miscible with increasing gas pressure. Finally, it becomes miscible by increasing the gas pressure from the minimum miscibility pressure (MMP: the pressure at which gas and oil create miscibility through the flow)^1–3^. In immiscible conditions, the fingering of gas increases due to the unfavorable viscosity ratio, and as a result, the oil recovery decreases. Under near-miscible conditions, microfluidic experiments have shown that a potential mechanism for recovering oil trapped behind the gas front is the cross-flow of oil and gas or diffusion^4^. Under miscible conditions, gas and oil can develop miscibility at first contact or multi-contact, and the movement becomes a single-phase flow^5,6^. Sweeping efficiency is low in all cases but improves with increasing miscibility^7^.

There are several experimental methods for MMP measurement, such as Slim Tube Test (STT), rising bubble apparatus (RBA), and vanishing interfacial tension (VIT) technique^8^. Among these, the Slim Tube Test (STT) has been widely used^9,10^ and has been accepted as the standard method of calculating the MMP^11^. Laboratory methods for determining MMP are costly and time-consuming (such as the Slim Tube Test) or cannot predict MMP systems with condensing/evaporative propulsion (such as rising bubble apparatus and multiple contact tests). However, they can generate useful phase behavior data to develop and validate MMP computational methods^12^.

Computational methods for MMP estimation have been developed over the years to use cubic equations of state for MMP estimation^13^. The basic assumption of all computational methods is that the phase behavior can be accurately described with a suitable cubic equation of state^14^. To accurately estimate the MMP, this assumption must be valid, especially near the critical region. There are three main computational methods: combined simulation of the slim tube, computational analysis with Method of Characteristic (MOC), and multiple mixing cell models. In the following, these methods are presented along with their advantages and disadvantages.

The Slim Tube simulation repeats the Slim Tube Tests (STT) in a computational simulator^15^. A combined simulator solves a one-dimensional flow equation using a cubic equation set of states for oil and gas (often without the term dispersion). The methodology and interpretation of the results are the same as the experimental methods. Therefore, the oil recovery curve in terms of pressure in 1.2 volumes of injected gas pores is considered. The breakpoint in 90% recycling can then be used as a criterion for MMP. Slim tube simulation is significantly cheaper and faster than actual experiments. However, for a reliable MMP estimate, the oil and gas-phase behavior must be well described using the state equation, which is especially true in the near-critical region. Slim tube simulation also has its drawbacks. First, it is slower and more time-consuming than other computational methods. Adjustment and calibration time is significantly longer than other computational methods. In addition, several simulations with different numbers of network blocks are required to estimate the MMP reliably^9^. In addition, MMP estimation using simulation can be affected by numerical dispersion because the slim tube simulation is based on the finite difference method^9,16^. Dispersion leads to loss of mixability, as it causes the composition path to enter the two-phase region. One way to minimize this effect is to use many network blocks. A more effective option is to run simulations for variable grid block sizes and then extrapolate oil recovery to infinite block sizes. This extrapolation is performed using the recovery plot in terms of 1/(Δx)^2^^17^ or 1/Δx^18^. Total variation diminishing schemes (TVD) can be used in the simulation to minimize the dispersion effect. A TVD scheme reduces adhesion and thus the dispersion effect^19^.

MMP estimation using the method of characteristic (MOC) refers to an algorithm obtained by solving one-dimensional fluid flow equations without dispersion^20^. MOC is a mathematical method for solving hyperbolic partial differential equations, such as one-dimensional fluid flow equations. The term MOC refers to an algorithm for estimating MMP based on a displacement analytical solution using the Method of Characteristic. The following is a review of the MOC method, its development, and its disadvantages.

**Figure 1:**
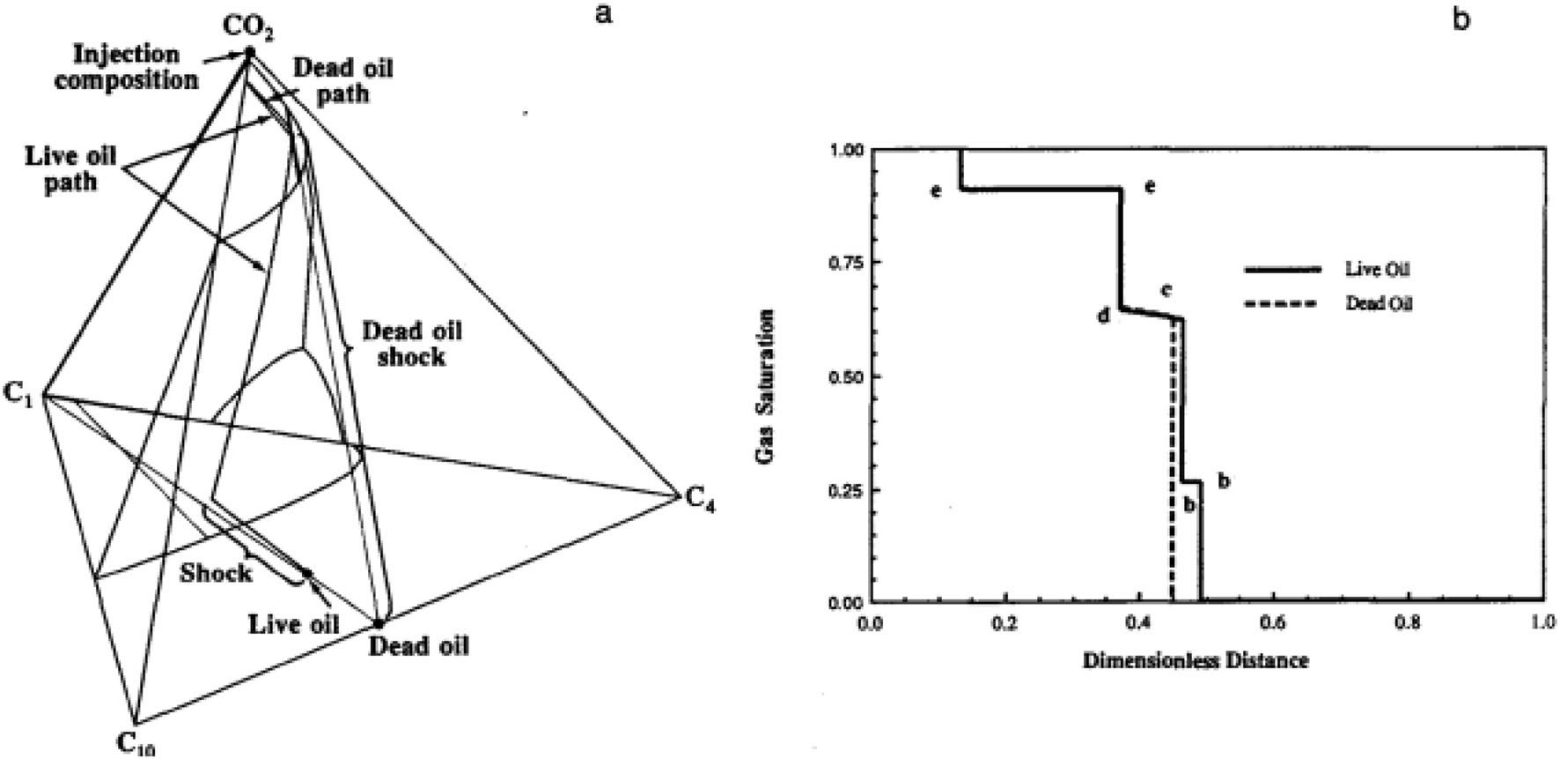
Evaluation of (**a**) composition paths and (**b**) saturation profiles for live and dead-oil displacements^19^.

Monroe *et al.*^21^ first examined the analytical theory for four-component systems and showed a third principal tie line in the movement path, called the cross tie line (Fig. 1).

Orr *et al.*^22^ and Johns *et al.*^23^ confirmed the presence of a cross-tie line for condenser/evaporator drives and provided a simple geometric structure for finding the main tie-lines. They showed that the MMP occurs at a pressure at which each of the three key tie lines first intersects the critical point (length becomes zero). In addition, Johns et al. showed that the crossing tie-line controls the development of miscibility in condensing/evaporating drives. Assuming a pure condensing drive or a pure evaporating drive, the estimated MMP is less than the predicted MMP.

Wang and Orr^24^ presented their multi-component approach by calculating the MMP of injected gases with more than one component. Using the Newton–Raphson design in hybrid space, they found the crossing points of the key tie lines. Jessen *et al.*^25^ improved the speed of the Wang and Orr method by incorporating Fugacity equations into Newton–Raphson. In some cases, MMP can be calculated by mixing cells. The mixing cell method involves one or more virtual PVT cells in which phase equilibrium calculations are performed. The main idea in single and multiple mixing cell methods is to mix (analytically) gas and oil in repeated contacts and thus create new equilibrium compounds.

In short, computational methods have drawbacks despite their ease of use. Slim tube simulation is slower than other computational methods, and its results can be affected by dispersion. Also, this laboratory method cannot measure the MMP between asphaltene oil and gas. The MOC-based tie line approach can converge to the wrong tie line and is prone to break. Mixing cell methods are not yet well developed. All of them are simple slim tube simulations and therefore prone to dispersion. Therefore, due to the cost and time-consuming nature of laboratory methods and the inconsistency between the values obtained with the existing relationships, the use of equations of state and thermodynamic relationships is a suitable and efficient option for predicting MMP values. The problem-solving algorithm, type of equations of state, injection gas composition, and their binary interaction are effective parameters in MMP calculations.

There are various models in different scientific sources to investigate the MMP with the equation of state. However, only a few researchers have studied the effect of gas injection into a reservoir using cubic equations of state^11,22,23^. Composite reservoir simulations based on equations of state used almost cubic equations of state such as Soave–Redlich–Kwong (SRK) and Peng-Robinson equations. Therefore, a logical option is to develop a modeling method based on these equations of state to predict the effect of gas injection on EOR.

Other capabilities of EoS in this field can be mentioned as estimation of MMP in porous media and estimation of MMP considering asphaltene deposition. The basis for calculating the MMP using the modified equations of state is to calculate the IFT. In this theory, the following concept is used: where the IFT of two phases tends to zero, it approaches the pseudo-mixed conditions, and where the IFT becomes zero, the two phases will merge^26^. Recent studies have shown that the porous medium alters IFT^25^. Therefore, changes in the MMP must also be considered if porosity conditions are applied.

In this research, the MMP of oil and gas is estimated using the modified Peng-Robinson equation. As mentioned in section "Methodology", this equation has been modified to consider the petrophysical conditions of the reservoir and the effect of the porous medium on the IFT and MMP. Therefore, parameters such as reservoir heterogeneity, the effect of fracture, and change of injected gas composition in different petrophysical conditions on the values of IFT and the MMP are investigated for the first time. At the moment, there is no laboratory method that gives the IFT in porous media. Therefore, the main goal of this study is to estimate the IFT in the porous medium. These parameters are investigated and analyzed in the modified state equation using the proposed new algorithm.

## Methodology

In this study, the Peng-Robinson equation of state has been utilized to calculate the vapor–liquid equilibrium^27^, and the improved Young–Laplace equation is considered to improve the capillary effect^28^ as follows:

1 $$p=\frac{RT}{v-b}-\frac{a\alpha }{v\left(v+b\right)+b(v-b)}$$

1-a $$a={a}_{c}\alpha \left({T}_{r},\omega \right)$$

1-b $${a}_{c}=0.45724\frac{{R}^{2}{T}_{c}^{2}}{{P}_{c}}$$

1-c $$b=0.07780\frac{R{T}_{c}}{{P}_{c}}$$

where, ${P}_{c}$ is the critical pressure $; {T}_{c}$ is the critical temperature; $\alpha \left({T}_{r},\omega \right)$ is an alpha function that depends on the reduced temperature ${T}_{r}$ and the central factor $\omega$. The Fugacity equations for the mixture are addressed as follows:

2 $$ln{\varphi }_{i}^{V}=-ln\left[{Z}_{V}-{B}_{V}\right]+\frac{{b}_{i}}{b}\left({Z}_{V}-1\right)+\frac{{A}_{V}}{2\surd 2{B}_{V}}\left[\frac{1}{a}\left(2\surd {a}_{i}\sum_{j=1}^{nc}{Z}_{j}\surd {a}_{i}\left(1-{\delta }_{ij}\right)\right)-\frac{{b}_{i}}{b}\right]ln\left[\frac{{Z}_{V}+{B}_{V}\left(1+\surd 2\right)}{{Z}_{V}+{B}_{V}\left(1-\surd 2\right)}\right]$$

3 $$ln{\varphi }_{i}^{L}=-ln\left[{Z}_{L}-{B}_{L}\right]+\frac{{b}_{i}}{b}\left({Z}_{L}-1\right)+\frac{{A}_{L}}{2\surd 2{B}_{L}}\left[\frac{1}{a}\left(2\surd {a}_{i}\sum_{j=1}^{nc}{Z}_{j}\surd {a}_{i}\left(1-{\delta }_{ij}\right)\right)-\frac{{b}_{i}}{b}\right]ln\left[\frac{{Z}_{L}+{B}_{L}\left(1+\surd 2\right)}{{Z}_{L}+{B}_{L}\left(1-\surd 2\right)}\right]$$

where the coefficients $A$ and $B$ for the liquid and vapor phases are defined as follows.

2-a $${A}_{V}=\frac{a{P}^{V}}{{\left(RT\right)}^{2}}$$

2-b $${B}_{V}=\frac{b{P}^{V}}{RT}$$

3-a $${A}_{L}=\frac{a{P}^{L}}{{\left(RT\right)}^{2}}$$

3-b $${B}_{L}=\frac{b{P}^{L}}{RT}$$

The Van der Waals mixing rule is applied to calculate the parameters a and b of the mixture.

4 $$a=\sum_{i}\sum_{j}{x}_{i}{x}_{j}{\left({a}_{i}{a}_{j}\right)}^{0.5}\left(1-{\delta }_{ij}\right)$$

5 $$b=\sum_{i=1}^{n}{x}_{i}{b}_{i}$$

where ${\delta }_{ij}$ is a binary interaction parameter other than $i$ and $j$. Also ${\delta }_{ij}={\delta }_{ji}$ and ${0=\delta }_{ii}={\delta }_{jj}$.

### Calculation of modified temperature and critical pressure

In nanopores, changing the critical properties of confined fluids is an inevitable problem^29^. The relative critical pressure and temperature changes can be calculated using the following equation based on the modified absorption Peng-Robinson equation^30^.

6 $$\Delta {T}_{c}=\frac{{T}_{c}-{T}_{cm}}{{T}_{c}}=0.679{\left({\sigma }_{LJ}/{R}_{P}\right)}^{0.7878}$$

7 $$\Delta {P}_{c}=\frac{{P}_{c}-{P}_{cm}}{P}=1.3588{\left({\sigma }_{LJ}/{R}_{P}\right)}^{0.7878}-0.4616{\left({\sigma }_{LJ}/{R}_{P}\right)}^{1.3588}$$

8 $${\sigma }_{LJ}=0.244\surd \frac{{T}_{c}}{{P}_{c}}$$

Here, $\Delta {T}_{c}$ and $\Delta {P}_{c}$ are the modified temperature and pressure due to confinement, respectively. T_c_ and ${T}_{cm}$ are the critical temperatures of bulk and nanopores, respectively, and ${P}_{c}$ and ${P}_{cm}$ are the critical pressures of bulk and nanopores, respectively. ${\sigma }_{LJ}$ is a Leonard-Jones size parameter.

### Phase equilibrium calculations by applying capillary pressure

9 $${f}_{i}^{L}\left(x,T,{P}^{L}\right)={f}_{i}^{V}\left(x,T,{P}^{V}\right)$$

10 $${x}_{i}{\varphi }_{i}^{L}\left(x,T,{P}^{L}\right){P}^{L}={y}_{i}{\varphi }_{i}^{V}\left(x,T,{P}^{V}\right){P}^{V}$$

The ${K}_{i}$ parameter is used in the Rachford-Rice method to obtain ${x}_{i}$ and ${y}_{i}$^31^. The initial ${K}_{i}$ is also obtained from the Wilson equation^32^.

11 $${K}_{i}=\frac{{P}_{ci}}{P}exp\left[5.37\left(1+{\omega }_{i}\right)\left(1-\frac{{T}_{ci}}{P}\right)\right]$$

12 $${K}_{i}=\frac{{y}_{i}}{{x}_{i}}=\frac{{\varphi }_{i}^{L}{P}^{L}}{{\varphi }_{i}^{V}{P}^{V}}$$

13 $$\sum_{i=1}^{{N}_{c}}\frac{\left({K}_{i}-1\right){z}_{i}}{1+\alpha \left({K}_{i}-1\right)}=0$$

Capillary pressure is defined as the difference between ${P}^{L}$ and ${P}^{V}$.

14 $${P}^{V}-{P}^{L}={P}_{cap}$$

15 $${P}_{cap}=\frac{2\sigma cos\theta }{{R}_{p}}$$

16 $$\sigma =\frac{{\sigma }_{\infty }}{1+2\frac{\delta }{{R}_{e}}}$$

where, ${R}_{p}$ is the pore radius, $\sigma$ denotes the IFT, ${\sigma }_{\infty }$ represents the flat IFT of the fluid, and $\theta$ is the contact angle. ${\sigma }_{\infty }$ can also be calculated by the Macleod-Sugden equation^33^.

17 $${\sigma }_{\infty }={\left[\sum_{i=1}^{{N}_{c}}{X}_{i}\left({x}_{i}{\rho }^{L}\left(T\right)-{y}_{i}{\rho }^{V}\left(T\right)\right)\right]}^{4}$$

where, ${x}_{i}$ and ${y}_{i}$ are the molar fractions of the liquid and gas phases obtained from the flash calculations, ${\rho }^{L}$ and ${\rho }^{V}$ are the molar densities of the liquid and gas phases, and ${X}_{i}$ is the Parachor parameter, respectively.

**Figure 2:**
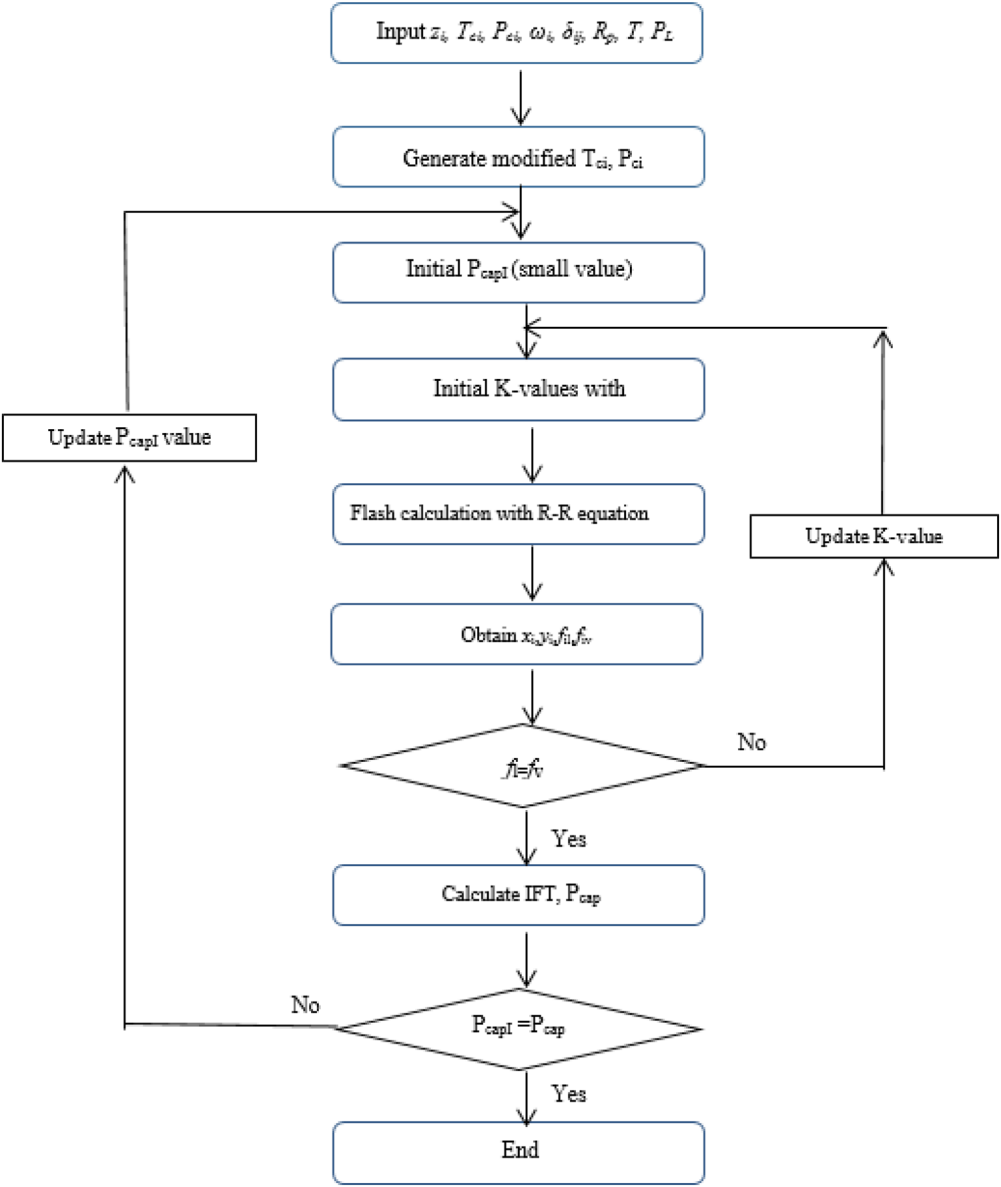
Flash and IFT calculation algorithm considering the capillary pressure.

The vapor–liquid-equilibrium (VLE or flash calculation) and IFT calculation algorithm considering the capillary pressure has been presented in Fig. 2. Enter the oil and gas inlet composition, temperature, pore size, and component properties in the first step. The second step calculates the changes in critical properties caused by being in a porous medium. Then a small initial value for the capillary pressure is guessed. Using the Rachford-Rice equation, the fugacity values and the molar fraction of the phases are calculated. The Wilson equation is used to initialize and update the *K*_*i*_ parameter. If the difference between the capillary pressure values of each stage and its previous stage is negligible, the calculations are completed, and the IFT value is reported. Otherwise, by updating the capillary pressure, the calculations will continue until the above conditions are reached.

To calculate the MMP at different pressures, the IFT values are calculated by the above algorithm, and the IFT is plotted in terms of pressure. The MMP occurs at a IFT close to zero.

## Discussion and results

In this section, the validation of the results is first examined using comparisons with experimental (laboratory) results in the references and are then examined in three different categories of injection of CH_4_, CO_2_, and N_2_ gases into nC_10_ as a representative of n-alkanes.

### Validation using IFT data of N_2_/nC_7_ system

**Figure 3:**
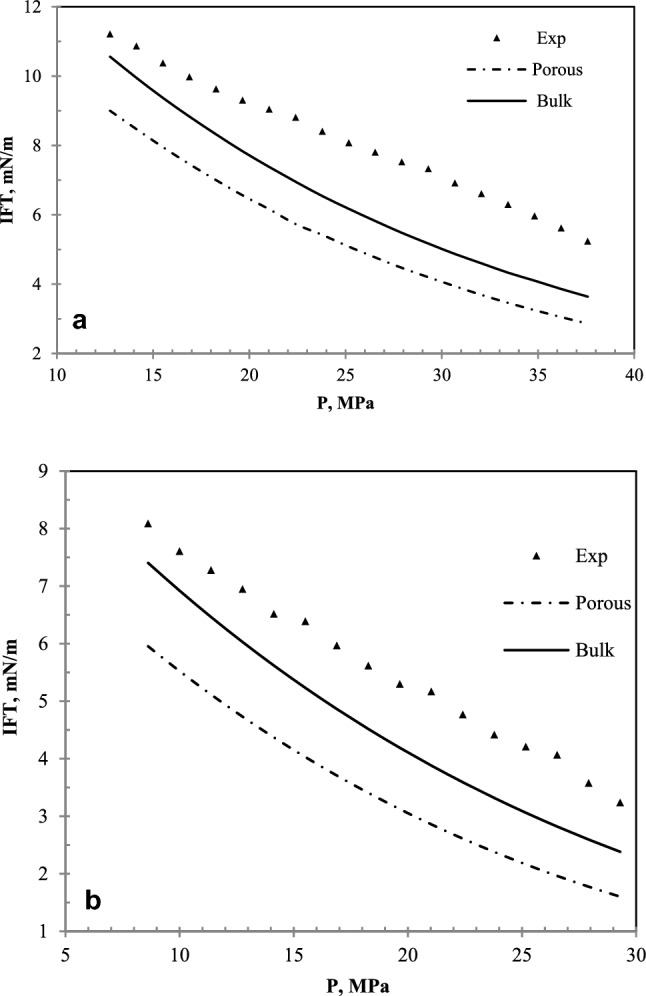
(**a**) IFT plot of N_2_ and nC_7_ in terms of pressure at the temperature of 313.15 K. Experimental data^34^. (**b**) IFT plot of N_2_ and nC_7_ in terms of pressure at the temperature of 393.15 K. Experimental data^34^.

Zolghadr et al.^34^, in an experimental study, investigated the effects of temperature, pressure, and paraffin groups on the miscibility of N_2_ in hydrocarbon liquids including nC_7_ using the IFT measurement method. These experiments were performed at five temperatures (313.15, 333.15, 353.15, 373.15, and 393.15 K) and a pressure range from 0.34 MPa to pressures close to miscibility. In this work, the laboratory data of the experiments related to the studies of Zolghadr et al.^33^ have been compared with the values obtained from the IFT modeling with the modified Peng-Robinson equation of state, and using the algorithm presented in this study at two temperatures of 313.15 and 393.5 K and presented in Fig. 3.

**Figure 4:**
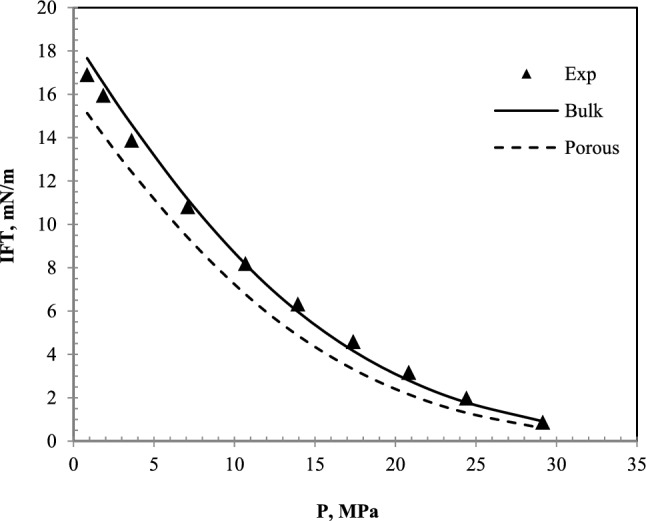
IFT plot in terms of pressure related to the nC_10_ and CH_4_ system at 343.15 K. Experimental data^35^.

The results show that the IFT decreases with pressure increment to finally reach the MMP at the IFT values close to zero. As can be seen in Fig. 3a,b, the changes in IFT in terms of pressure are not entirely linear. The closer they are to higher pressures, the lower the slope of the IFT decreases. These changes are due to the nature of N_2_. In their laboratory studies, Kazemzadeh et al. obtained similar results regarding the IFT values of the N_2_-hydrocarbon system^1^. They presented the IFT plot in terms of pressure as a quadratic function. Figure 4 shows the IFT plot in terms of pressure for different states. The laboratory data shown in the plot are taken from the research work of Zolghadr *et al.*^33^. In these data, due to the nature of laboratory conditions, fluctuations in terms of pressure are observed, which is expected due to laboratory error. However, the other two plots, namely bulk and porous medium states, are extracted from the used model in this paper. The bulk case is when IFT is calculated in a system outside the porous medium. The porous state is also considered a porous medium with a porosity of 10% and a permeability of 10 mD. According to the results, it can be found that the IFT calculated at any given pressure is less in the porous medium than in bulk. Therefore, it is clear that the MMP calculated in the porous medium is less than the bulk medium, and both states have lower values than the results obtained by Zolghadr *et al.*^33^. Lower IFT and consequently the MMP in the porous medium are operationally essential. If the MMP of the two phases of oil and gas decreases in the porous medium, it indicates that the miscibility takes place at a lower pressure, and we will reach the mixing condition sooner. Therefore, in water-based enhanced recovery, it should be considered that the presented values of the MMP obtained by laboratory measurement of IFT can be lower than the case of the porous medium. Therefore, it is necessary to consider the effect of the porous environment using models such as the model presented in this study. Table 1 calculates the error of the results obtained from the IFT of the N_2_/nC_7_ system of the model (in the bulk state) with laboratory results at five different temperatures.

**Table 1:** The calculated error of IFT for the system of nC_10_–N_2_ at different temperatures in the bulk state.

Media	AARD%				
Bulk	Temperature (K)				
	393.15	373.15	353.15	333.15	313.15
	19.98	17.92	18.1	25.62	5.80

The IFT error has been calculated from the following equation and has been presented in Table 1.

17 $$AARD\%=\frac{100}{N}{\sum }_{1}^{N}\left|\frac{{X}_{i}^{exp}-{X}_{i}^{Cal}}{{X}_{i}^{exp}}\right|$$

In the worst case, the measured error is less than 19.98%, which occurs at high temperatures (i.e. 393.15 K). Here, the model results in the bulk state were compared with the experimental results and showed a small error. However, the main feature of this model is to provide IFT and the MMP in the porous medium, which provides valuable results in this regard. The model presented in this study has the ability to calculate IFT at any temperature and pressure. IFT is measured in the laboratory in different pressure ranges, but at high pressures, due to the phenomenon of VIT (Vanishing Interfacial Tension), it cannot perform this measurement. To measure the MMP, the IFT data should be extrapolated in terms of pressure to reach zero IFT. One of the advantages of the presented model is that it can give the IFT at high pressures, which helps a lot in the accurate estimation of the MMP.

### MMP in CH_4_ gas injection: IFT in nC_10_/CH_4_ system

Cumicheo et al.^35^ modeled the IFT in binary systems of CH_4_, CO_2_, N_2_, and alkanes to obtain the experimental data used from several papers with equations of state and mathematical models. In this research, experimental data of CH_4_, CO_2_, N_2_, and nC_10_ systems have been examined. The IFT values in both bulk and porous media are calculated using the Peng-Robinson equation and porous media equations.

Figure 4 shows the IFT plot in terms of pressure related to the nC_10_ and CH_4_ system at 343.15 °K.

Figure 4 represents an interesting result. This figure shows that the values of IFT of the CH_4_ and nC_10_ system in the porous medium are less than the bulk condition. However, the difference in values at higher pressures decreases, which means that as the pressure increases, the difference between the values of IFT in the bulk state and the porous medium will be negligible. So that in pseudo-miscible and miscible conditions, this difference can be ignored. The difference in the porous and bulk media results in these two cases can be due to the activation of vaporizing and condensing mechanisms. This means that a porous medium and increasing the contact surface of oil and gas at low pressures cause these two mechanisms to be more active simultaneously and reduce the IFT compared to the bulk state. But at high pressures, this effect is almost diminished.

**Figure 5:**
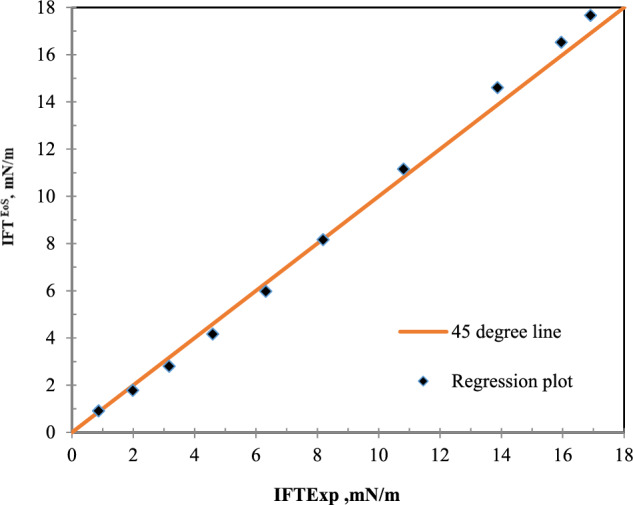
A comparative plot of laboratory^35^ and calculated values of IFT with the equation of state in the bulk state for nC_10_ and CH_4_ system at 343.2 K.

Figure 5 has been plotted to compare the laboratory results and model results in the bulk state. According to Fig. 5, the model results and the laboratory results are in good agreement in this system.

The first understood concept from this section is that this model will be able to accurately predict the IFT of the hydrocarbon and CH_4_ systems. Figure 5 shows that the results of the model are in good agreement with the laboratory results.

The equilibrium of the phase fluid in a dense porous medium is disturbed due to capillary pressure in nanopores containing organic matter. The phenomenon of mixing between gas and oil also changes during gas injection to improve oil recovery. Therefore, it is necessary to develop a general framework of theoretical models and algorithms to investigate the effect of pore proximity on the phase behavior and fineness of finite liquids in pores.

### MMP in CO_2_ gas injection: IFT in nC_10_/ CO_2_ system

**Figure 6:**
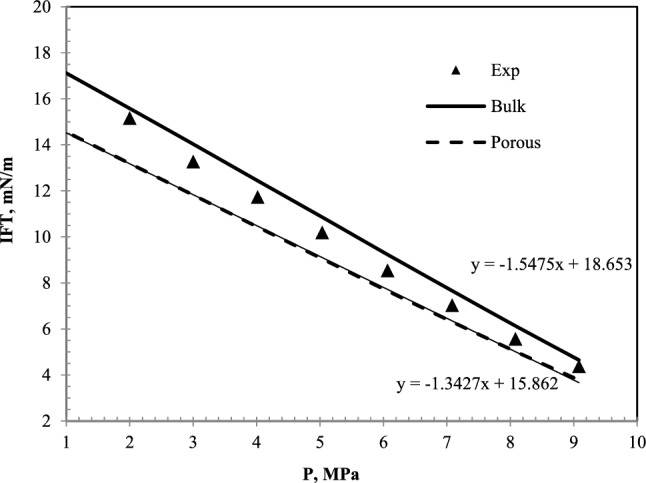
IFT plot of nC_10_and CO_2_ system at 343.55 K and different pressures. Experimental data^35^.

Figure 6 shows the IFT data in terms of pressure for the nC_10_ and CO_2_ systems at different pressures. This figure shows three sets of data, including laboratory data extracted from Cumicheo et al. and Lake^34,35^, IFT data in bulk medium, and IFT data in the porous medium.

**Figure 7:**
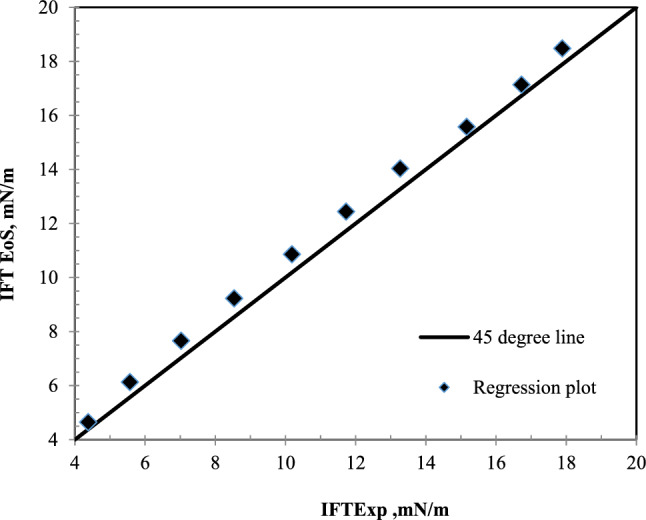
A comparative plot of laboratory^35^ and calculated IFT with the equation of state for nC_10_ and CO_2_ system at 343.55 K.

As shown in Fig. 6, the laboratory data differ slightly from the IFT data in the bulk medium. The data extracted from this model and the laboratory results in Fig. 7 have been presented to determine the differences.

It is still observed that, like CH_4_ injection, the data on the porous medium are less than the bulk medium. The IFT reduction with increasing pressure in all three data sets is almost linear. This means that the IFT can be achieved by extending this straight line. The difference between the bulk data and the porous medium decreases with increasing pressure, but they still have a difference of about half a mN/m at a pressure of 9 MPa. The slope difference between the IFT data in the bulk and porous media causes the MMP to be affected. The slope of the IFT plot in terms of pressure in the porous medium is less than the slope of the plot in the bulk state. Therefore, despite this slope difference, because the values of IFT in the porous medium are lower at low pressures, the extension of these graphs causes the difference between the MMP in these two states is lower than what is felt in the measurement at initial pressures. In other words, it is observed that with each megapascal of system pressure, the IFT in the bulk medium decreases with a linear trend of 1.55 mN/m. While in porous media, this decrease is 1.34 mN/m. The MMP is 12.05 MPa in the bulk medium and 11.81 MPa in the porous medium. This is in the context that the difference in IFT at ambient pressure in both bulk and porous states is 2.79 mN/m.

### MMP in N_2_ gas injection: IFT in nC_10_/ N_2_ system

**Figure 8:**
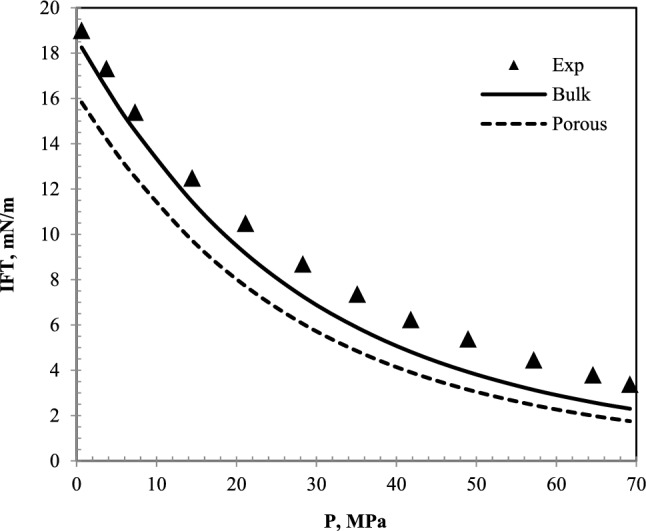
IFT plot of nC_10_ and N_2_ system at 343.2 K and different pressures.

**Figure 9:**
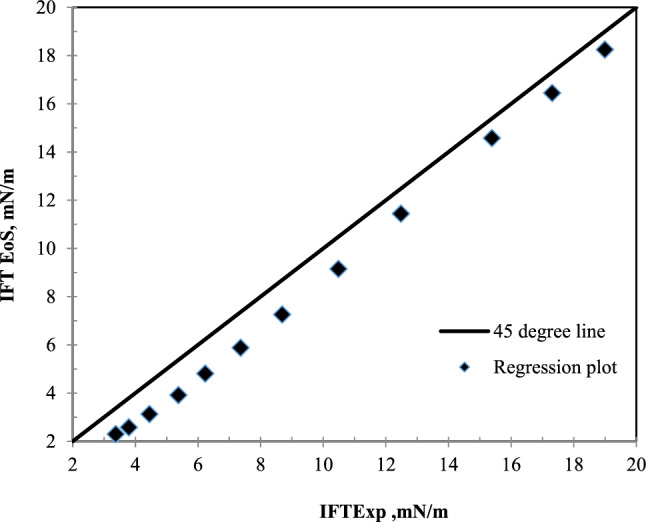
A comparative plot of laboratory^35^ and calculated IFT with the equation of state for nC_10_ and N_2_ system at 343.2 K.

The IFT data of the nC_10_ and N_2_ gas system at different pressures have been plotted in Fig. 8. According to this figure, the laboratory data^35^ study are slightly different from the data presented in this model. Figure 9 has been plotted to know the data deviation.

According to Figs. 8 and 9, the experimental data and the data provided by the model at low pressures are almost consistent. However, the difference between the IFT of the laboratory and this model increases with increasing pressure. The maximum difference is about one mN/m. The difference in these values at high pressures can be affected by the measurement error in equilibrium in the high-pressure laboratory method.

Concerning the difference between bulk and porous medium plot, similar to CO_2_, CH_4_ and N_2_ gas, it is observed that the IFT in the porous medium is always less than in the bulk medium. As mentioned earlier, the reduction of IFT in terms of pressure in the N_2_ gas injection system and the nC_10_ is not linear. It decreases with the trend of a quadratic function. The decreasing trend of IFT in terms of pressure has been previously studied and confirmed in the studies of Doryani *et al.*^36^. The values of IFT error for nC_10_ systems in the presence of different gases of CH_4_, CO_2_, and N_2_ at different temperatures are shown in Table 2.

**Table 2:** Error calculated for CH_4_–nC_10_, CO_2_–nC_10_ and N_2_–nC_10_ systems at different temperatures.

System	AARD% Temperature (K)					
	313.3	343.2	323.35	343.55	313.15	343.2
CH_4_–nC_10_	9.32	5.80	–	–	–	–
CO_2_–nC_10_	–	–	13.60	6.01	–	–
N_2_–nC_10_	–	–	–	–	19.11	17.95

The results show that the model for the two CH_4_/nC_10_ and CO_2_/nC_10_ systems is in good agreement with the experimental data. However, it does not predict the IFT of N_2_ in the presence of nC_10_. The error occurs at lower temperatures and higher pressures.

### The effect of mean pore radius on IFT

**Figure 10:**
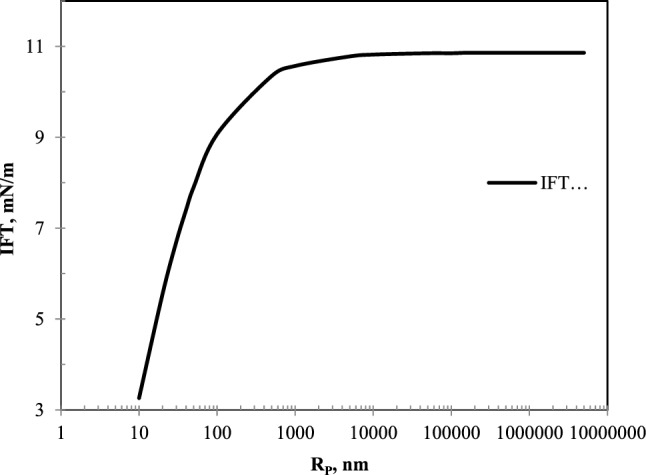
IFT plot in terms of Rp for nC_10_/CO_2_ system at 323.35 K and 5 MPa pressure.

The IFT plot in terms of pore radius has been presented in Fig. 10 to investigate the effect of pore radius on IFT in the nC_10_ and CO_2_ system at constant temperature and pressure. As shown in the figure, the plot has two slopes. At low values of Rp, with increasing pore radius, the IFT increases with a high slope. While entering the bulk region, the slope of changes decreases with increasing Rp value and is almost constant.

In other words, according to Fig. 10, it can be deduced that in very small pores, IFT is significantly reduced. However, if the pore diameter exceeds a certain threshold, the pore size does not affect the IFT. This is especially important in fractured reservoirs where the pore diameters vary greatly from matrix to fracture. In short, in a specific thermodynamic condition, it causes that the IFT is more in the fractures, and this quantity is less in the matrix. Therefore, the presence of IFT variation profile causes that in addition to vaporizing and condensing mechanisms, a phenomenon such as Marangoni also causes the fluid to move from the porous medium to the fracture or vice versa and control oil recovery.

## Conclusion

According to the results obtained in the modeling, the following conclusions can be mentioned:

- The proposed model can calculate the IFT between hydrocarbon gases, CO_2_, and n-alkanes in various thermodynamic conditions showing reasonable accuracy in bulk conditions. It can be an excellent alternative to time-consuming and costly laboratory methods. This model estimates the MMP in the injection of hydrocarbon gas and CO_2_ gas into n-alkanes.
- One of the most important problems in industry is the inconsistency of the MMP calculated from the vanishing interfacial tension (VIT) technique and reality in the reservoir. This model will be able to apply the effect of the porous medium (mean pore radius) on the calculation of the MMP. By modifying the equation of state, the model will be able to provide the values of IFT in a porous medium (in a state equivalent to the mean pore radius) and calculate the MMP by extrapolating the IFT data in terms of pressure.
- As the mean pore radius increases, the IFT increases with a significant slope. However, from a certain radius, the interstice size does not affect the values of IFT. This indicates that the MMP obtained in the bulk medium (using the VIT technique) in the laboratory for a fractured reservoir confirms the MMP and the IFT values in the fracture. To calculate the IFT in the matrix, it is necessary to plot the IFT values as a function of the mean pore diameter using modeling.
- Heterogeneity of the reservoir causes the profile of IFT changes in it. The formation of this phenomenon causes a phenomenon such as Marangoni and causes the movement of fluid and affects the oil recovery by controlling the forward front of the fluid while increasing the mass transfer between the two fluids.

## Data Availability

All data generated or analysed during this study are included in this published article.

## List of symbols

a, b: EoS constants
A, B: Liquid and vapor phase coefficients
K_i_: Vapor − liquid equilibrium ratio of component i
${\delta }_{ij}$: Binary interaction parameter
MW: Molecular weight (g/mol)
N: Total number of data
P: Pressure (MPa)
P_C_: Bulk critical pressure (MPa)
P_cm_: Nanopore critical pressure (MPa)
$${X}_{i}$$: Parachor constant
R: Universal gas constant
T: Temperature (K)
T_C_: Bulk critical temperature (K)
T_cm_: Nanopore critical temperature (K)
T_r_: Reduced temperature
V: Molar volume (m^3^/mol)
x: Liquid fraction
y: Vapor fraction
Z: Compressibility factor
ω: Acentric factor
φ: Fugacity coefficient
*f*: Fugacity
*ρ*: Molar density
$\Delta {T}_{c}$: Modified temperature due to confinement
$\Delta {P}_{c}$: Modified pressure due to confinement
${\sigma }_{LJ}$: Leonard-Jones size parameter
${P}_{cap}$: Capillary pressure (MPa)
$${R}_{p}$$: Pore radius
R_e_: Effective radius, m
$$\sigma$$: Interfacial tension
${\sigma }_{\infty }$: Flat interfacial tension of the fluid
δ: Thickness of adsorption region, m
$$\theta$$: Contact angle

## Abbreviations

PVT: Pressure volume temperature
AARD: Average absolute relative deviation
IFT: Interfacial tension
MMP: Minimum miscibility pressure
EoS: Equation of state
STT: Slim tube test
VIT: Vanishing interfacial tension
RBA: Rising bubble apparatus
TVD: Total variation diminishing
MOC: Method of characteristic
EOR: Enhanced oil recovery
PR: Peng-Robinson
SRK: Soave-Redlich-Kwong
